# Does Caregiver Engagement Predict Outcomes of Adolescent Wilderness Therapy?

**DOI:** 10.3390/bs15010054

**Published:** 2025-01-08

**Authors:** Joanna E. Bettmann, Naomi Martinez Gutierrez, Annelise Jolley, Laura Mills

**Affiliations:** 1 College of Social Work, University of Utah, 395 South 1500 East, Salt Lake City, UT 84112, USA; annelise.jolley@utah.edu; 2 Department of Psychology, York University, 4700 Keele Street, Toronto, ON M3J 1P3, Canada; naomimg@yorku.ca; 3 Pine River Institute, 180 Dundas Street West, Suite 1410, Toronto, ON M5G 1Z8, Canada; laura.m@pineriverinstitute.com

**Keywords:** adolescent treatment, wilderness therapy, family therapy, adolescent mental health, outdoor behavioral healthcare

## Abstract

Existing research shows some links between wilderness therapy outcomes and familial functioning. However, wilderness therapy programs do not agree on what kind of caregiver involvement is required to improve adolescent program outcomes, nor has research examined different types of family engagement and their impact on adolescent treatment outcomes. Thus, the present study explored the research question: Does caregiver engagement in adolescent wilderness therapy foster improved outcomes? The study sample consisted of 4067 adolescent wilderness therapy clients from 12 different wilderness therapy programs. Using standardized measures and multilevel structural equation modeling, the authors found that caregiver program participation significantly predicted adolescent mental health outcomes of the program, suggesting that the more caregivers were involved in family interventions during the program, the more likely their adolescent child was to improve in the program. The study also found that greater caregiver effort predicted greater mean change in adolescent mental health outcomes of wilderness therapy. This study suggests the importance of enhancing familial interventions in adolescents’ wilderness therapy programs in order to improve adolescent outcomes. Given findings from this study, wilderness therapy programs should consider expanding the ways that they involve families in treatment in order to optimize adolescent outcomes.

## 1. Introduction

Wilderness therapy (WT) is a short-term residential treatment that utilizes outdoor expeditions, group living, self-reflection, and various therapeutic formats in a nature-based setting (Bettmann et al., 2016). Most programs offer a combination of individual therapy, group therapy, and physical and mental challenges (Bettmann & Tucker, 2011; Bettmann et al., 2017; Hoag et al., 2013; Tucker et al., 2022).

Research indicates positive outcomes for adolescents in WT (Bettmann et al., 2016; DeMille et al., 2018), including decreased substance use and improved emotional and behavioral functioning (Bettmann et al., 2013; Tucker et al., 2014). Many WT participants experience significant improvements between intake and discharge, as reported by parents and adolescents on measures such as the Youth Outcomes Questionnaire (Harper et al., 2019; Tucker et al., 2016a). In one study comparing adolescent outcomes in outpatient treatment and WT, both groups improved clinically and statistically (DeMille et al., 2018). Notably, those who participated in WT and returned home after treatment showed greater improvement one year post-treatment compared to the adolescents who received outpatient treatment only, suggesting that the strength and durability of adolescent symptom improvement from WT (DeMille et al., 2018).

Recent research suggests that some adolescents may deteriorate or emerge unchanged from WT participation (Bettmann et al., 2023). Several variables appear to influence adolescent WT outcomes in both positive and negative directions, including length of stay (Magle-Haberek et al., 2012), client demographics (Tucker et al., 2014; Combs et al., 2016a), presenting problem (Tucker et al., 2014), use of transportation services (Harper et al., 2021), and level of family involvement (Harper & Russell, 2008). Hence, research should unpack the influences that help or hinder progress for WT participants. Although there is extant research that identifies both positive and negative influences in WT outcomes, little research examines the role of caregivers in adolescent outcomes.

### 1.1. Client Demographics as Variables in WT Outcomes

Multiple studies report that female-identified adolescents enter WT programs with higher levels of distress and report greater clinical change than their male-identified counterparts (Bettmann et al., 2023; Combs et al., 2016b; Tucker et al., 2014). In comparison, gender-diverse adolescents both start and finish WT with higher levels of distress (Bettmann et al., 2023). In one study, gender was a significant predictor of outcomes, with females reporting larger improvements in WT; this finding is notable as there was no significant difference between intake scores and length of treatment between males and females (Tucker et al., 2014). This finding may connect to the empowering nature of WT for some clients; additionally, WT aims to provide participants with a sense of confidence and mastery, which may help explain this finding for females (Tucker et al., 2013).

Research examining adolescent residential treatment outcomes finds that older clients are “more likely to successfully ‘recover’ in the [residential treatment center] environment compared to younger clients” (Magle-Haberek et al., 2012, p. 214). Similarly, research suggests that WT outcomes for older adolescents are better than those for younger adolescents (Bettmann et al., 2023). Adoptive status may also affect WT outcomes: adopted adolescents enter WT and residential treatment with a higher degree of suicidality, a higher likelihood of experiencing recent trauma, and higher levels of mental illness or substance abuse in their family history (Bettmann et al., 2015). Adopted adolescents also may be more likely to deteriorate following WT compared to non-adopted adolescents (Bettmann et al., 2023; Combs et al., 2016b). While client demographics may explain some variability in treatment change, theory and research on family factors may illuminate why some clients change differently.

### 1.2. Family Systems in WT

Family systems theory posits that each family member affects all other members (W. P. Russell, 2019). From this theoretical perspective, programs may conceptualize individual symptomology as arising from the context of the family system and linked inextricably to the broader family functioning (Ferenchick & Rosenthal, 2019; Yahav & Sharlin, 2002). From the family systems perspective, adolescents’ symptoms have meaning within the family context and are understood best by examining and treating family dynamics.

Although some WT programs focus primarily on the adolescent as an individual, most contemporary WT includes family therapeutic work: 73% of private pay adolescent treatment programs and 32% of programs for adjudicated adolescents incorporate some family programming (Smith & Issenmann, 2017). These interventions range from assigned readings to weekly family therapy meetings to in-person seminars for parents. There is a wide variety of family interventions and family therapy focus between programs (Smith & Issenmann, 2017; Tucker et al., 2016b). A mixed-methods study exploring the impact of WT on family functioning found that families who conceptualized WT through a “child-as-client” lens rather than a “family-as-client” (family systems) lens were less likely to experience long-term improvement in functioning (Harper & Russell, 2008, p. 31). In one study, parents of adolescent clients explained that their adolescent children could not be responsible for making changes in their parents or families (Cohen & Zeitz, 2016).

Adolescents often make more progress towards therapeutic goals than their parents during WT due to participating in therapeutic work for more hours each day. However, most programs recommend that parents be as participatory as possible to improve adolescent outcomes and help their children feel supported despite being far from home (Cohen & Zeitz, 2016). Many adolescents’ families live a significant distance from the treatment facility. In a case study of two families with an adolescent in WT, parents identified that the geographical distance allowed for more positive change in the family system at home, including time to learn new therapeutic strategies and participate in psychoeducation (Cohen & Zeitz, 2016). Some WT programs integrate family interventions because many believe that change in the family at home is the strongest predictor of long-term success after the adolescent returns from WT (Harper & Russell, 2008).

### 1.3. The Connection Between Family Functioning and Wilderness Therapy Outcomes

Adolescents’ WT outcomes correlate with family functioning, as better family functioning predicts better adolescent WT outcomes (Liermann & Norton, 2016; Tucker et al., 2016a). Notably, negative family dynamics correlate with higher attrition rates in WT, suggesting a need for focus on family functioning (Liermann & Norton, 2016). Families who make the most changes while their adolescent child is at WT have better family outcomes (Harper & Russell, 2008). One case study of adolescent boys who participated in WT concluded that families may become closer and repair relational wounds resulting from changes in family dynamics while the adolescent child is in WT (Cohen & Zeitz, 2016). A different case study reported a stronger adolescent-parent relationship by the end of WT (K. C. Russell, 2000). Improved family dynamics link to lower levels of substance use and improved school performance following WT (Liermann & Norton, 2016).

### 1.4. Defining Caregiver Engagement

Caregiver engagement in WT correlates with improved adolescent outcomes, improved treatment engagement during WT, and increased parental efficacy after WT (Coll et al., 2018; Israel et al., 2007; Liermann & Norton, 2016). Caregiver engagement may be integral to the success of WT clients by helping to foster whole family system shifts. But existing research does not have an agreed-upon definition of caregiver engagement in WT (Smith & Issenmann, 2017).

One study defines caregiver engagement as having at least one biological parent as a passive presence in their adolescent child’s treatment sessions (Cardy et al., 2020), while another study defines it as parental involvement in treatment in any capacity (Coll et al., 2018). Caregiver engagement is defined as “mothers’ behavioral involvement and personal emotional involvement” in their adolescent’s treatment (Israel et al., 2007, p. 138) and as a “parent’s active, independent, and responsive contribution to treatment” (Haine-Schlagel & Walsh, 2015, p. 134).

Within WT research, one study defines caregiver engagement as having five components (Harper & Russell, 2008). These are mandatory parental involvement, inclusion of family goals in treatment plans, counseling/psychoeducation with families, remote family contact such as having the adolescent contact parents via letter writing, and parent-therapist phone calls or other types of direct parent involvement (Harper & Russell, 2008). This broader definition of caregiver engagement spans across multiple program elements of adolescent WT and thus serves as an appropriate definition for the present study.

### 1.5. The Present Study

Existing research shows connections between wilderness therapy outcomes and familial functioning (Coll et al., 2018; Harper & Russell, 2008; Liermann & Norton, 2016). However, programs do not agree on what specific caregiver involvement is required to improve adolescent WT outcomes. Thus, the present study explored the research question: Does caregiver engagement in adolescent WT foster improved outcomes?

## 2. Materials and Methods

### Participants

The study included participants from WT programs who were members of the National Association of Therapeutic Schools and Programs Practice Research Network (Tucker et al., 2016a). Member programs administered survey packages to clients and their caregivers within seven days of program admission and to clients, caregivers, and staff within seven days of discharge. Data were submitted electronically to a central database managed by the Outdoor Behavioral Healthcare Center at the University of New Hampshire. The Center provided data to the researchers. Data were anonymized at the individual client and program levels; thus, treatment frameworks, therapeutic approaches, length of stay, location, and other program elements could not be defined for the present study.

The sample consisted of 4067 adolescent WT program clients from 12 programs who attended WT between 2017 and 2023. Within the dataset, the number of clients per program ranged from *n* = 159 to *n* = 628. Only adolescents who consented (if 16 or over) or assented with parental consent to contribute to the research were included. The University New Hampshire Institutional Review Board approved the study before data collection. The University of Utah Institutional Review Board approved the present study prior to data analyses.

Adolescents in the study sample (*n* = 4067) were an average age of 15.9 years (*SD* = 1.32) at admission. The majority of clients were White (60.5%, *n* = 2460) and affluent, most (54%, *n* = 1631 of *n* = 3019, or 74.2% of respondents) coming from homes with family income over $200,000 per year. Only 18.4% (*n* = 554) came from a home with an income of less than $100,000. Most adolescents (83.2%; *n* = 3385) responded to the admissions survey question asking about gender identity. Of these, 59% (*n* = 1996) of adolescents identified as male, and 32.5% (*n* = 1099) identified as female. Adolescents who did not self-identify as female or male were coded in the present study as “gender diverse” (8.6%, *n* = 290). Of the caregivers that responded to the admissions survey question asking about adoption (75.9%; *n* = 3085), approximately 20% (*n* = 610) indicated their adolescent child was adopted. Clients stayed an average of 80.8 (*SD* = 41.7) days in the programs.

## 3. Measures

The study utilized a secondary dataset. Thus, the health indicators and measurement instruments were selected years before this study was conceived. The indicators selected by the authors of the present study include adolescent mental health, gender identity, age, and adoptive status, as well as adolescent perception of family functioning and feeling that they belonged at the treatment program. Also included in the present study were clinician ratings of parental effort and reports of caregiver participation in the therapeutic process. This collection of indicators was chosen as the most relevant to explore the relationship between caregiver engagement and youth treatment outcomes.

### 3.1. Adolescent Mental Health

The researcher examined changes in adolescent mental health, as measured by the Youth Outcome Questionnaire Self-Report 2.0 (YOQ-SR: Ridge et al., 2009; Wells et al., 2003; Wells et al., 2007). The YOQ-SR is a 64-item Likert-type assessment of adolescent functioning and includes the following subscales: intrapersonal distress (measuring depression, anxiety, fearfulness, etc.), somatic problems (measuring physical or somatic concerns), interpersonal relations (measuring difficulty with relationships), social problems (measuring behaviors such as aggression, defiance, and conflict), behavioral dysfunction (measuring difficulty with concentration, attention, or impulsivity), and critical items (such as suicidal ideation, self-harm, hallucinations, etc.). The YOQ is widely used and has well-established reliability (0.96) and validity (Ridge et al., 2009).

### 3.2. Gender Identity, Age, and Adoptive Status

The admission questionnaire to adolescents included: Which of the following choices best describes your gender identity? Response options were: male, female, transgender, gender fluid, I identify as (please specify)____, and I am not sure. Gender identity was coded as male, female, and gender diverse. Age at the time of admission was calculated based on date of birth and first day in the program. Whether or not each participant was adopted was collected at intake as a part of parent report from each WT program.

### 3.3. Belonging

The admission questionnaire also included, ‘It makes sense for me to be at this program’ with a sliding bar for ratings from 0 (strongly disagree) to 10 (strongly agree).

### 3.4. Family Functioning

The McMaster Family Assessment Device, General Functioning Subscale, Youth-report (FAD) is a 12-item Likert-type assessment of acceptance and agreeableness. The FAD is widely used and has >0.70 reliability (Ryan et al., 2006) and good validity (Sperry, 2012). The North American “healthy” family scores range from zero to two, with higher scores indicating greater dysfunction (Ryan et al., 2006). The FAD was administered to adolescent clients at program admission.

### 3.5. Clinician Observation of Parent Effort

Clinicians at each WT program completed discharge surveys for each adolescent, which included the following question: *Please rate the parents’ personal effort in their therapeutic work at home or on their own*. Response options were: *None*, *Low*, *Moderate*, *High*, and *Exceptional*.

### 3.6. Caregiver Program Participation

While WT programs in the study sample utilized a range of family therapy interventions (see Table 1), all programs provided weekly, facilitated, therapeutic family phone calls and on-site caregiver visits. Most clinicians (78.9%; *n* = 3385) responded to the discharge survey question asking about the number of therapeutic contacts with the caregivers. Of these responders, most had weekly therapeutic contacts with adolescents’ caregivers (78.9%; *n* = 3385). Most clinicians spent an hour in family therapy per contact (84.3%; *n* = 2706); only 2.3% (*n* = 75) spent 30 min; 11.2% (*n* = 360) spent one hour and 30 min; 1.3% (*n* = 42) spent two hours; 0.31% (*n* = 10) spent more than two hours; and 0.56% (*n* = 18) did not have therapeutic contacts with the caregivers.

Clinicians at each WT program reported on the time spent during each therapeutic call, the number of caregiver visits to the program, and the duration of each caregiver visit. Thus, the researchers decided to utilize these most common family therapy interventions to gauge the level of caregiver involvement in adolescent WT. The researchers conceptualized these three indicators (time spent during each therapeutic call, the number of caregiver visits to the program, and the duration of each caregiver visit) as a latent variable: caregiver program participation.

## 4. Analyses

### 4.1. Clinically Relevant Standardized Scores

Since YOQ subscales have different value ranges and clinically normed benchmarks, YOQ subscale scores were converted to a Clinically Relevant Standardized Score (CRSS). CRSS calculations were based on the clinical cutoff and Reliable Change Index (RCI) scores as recommended in the YOQ instrument manual (Burlingame et al., 2005). For example, in the subscale for anxiety and depression, scores range from −4 to 68. Scores of 17 or greater are considered in the clinically unhealthy range, and the RCI is 9. The CRSS was derived by subtracting the clinical cutoff value (17) from the client raw score and dividing the result by the RCI (9). ($\frac{x-CUTOFF}{RCI}$). A raw score of 36, for example, would be converted to a CRSS of 2.1; in this example, 2.1 indicates 2.1 clinically meaningful units less healthy than the clinical cutoff.

The CRSS indicates that 1 represents a clinically meaningful unit. A CRSS of 1 represents one clinically meaningful unit more symptomatic than the clinical cutoff of 0. A CRSS of −2 represents two clinically meaningful units *more* healthy than the clinical cutoff. Any change of 1 (or more) is a clinically relevant change. Changes on all outcome indicators were calculated by subtracting discharge from admission CRSS units.

### 4.2. Multilevel Structural Equation Modeling

**Figure 1:**
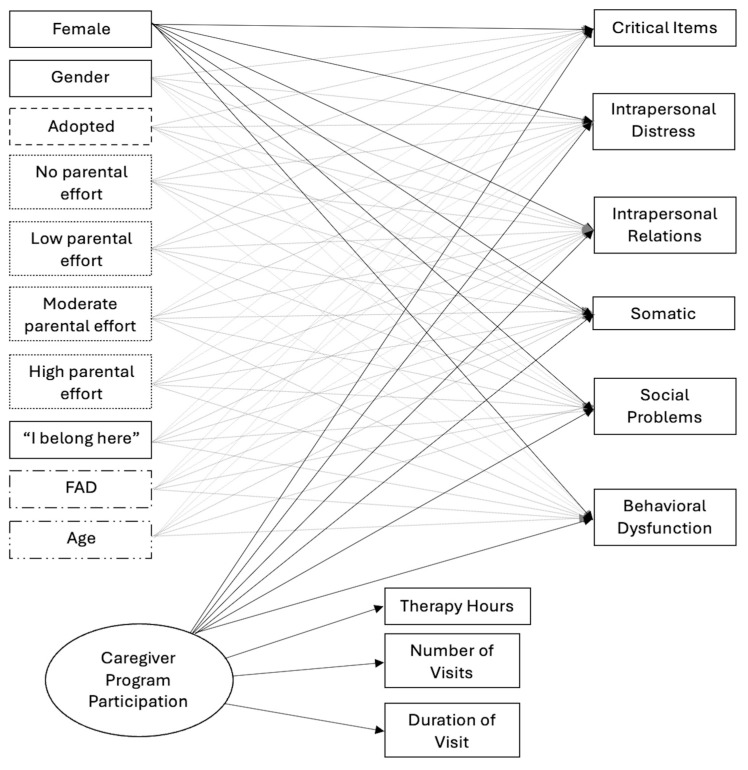
Hypothesized multilevel structural equation model.

A two-level, random-intercepts, multilevel structural equation regression modeling (MSEM) was estimated using full maximum likelihood in Mplus (Version 8). (See Figure 1). As a preliminary step, intraclass correlations between outcome subscales and Facility ID were conducted to understand if health changes were attributable to the program. The coefficients between facility ID and YOQ-SR subscales averaged 10%, suggesting substantial variance in mental health at different facilities. Facility ID was used as the grouping variable.

The researchers used structural equation modeling to estimate a latent construct for Caregiver Program Participation (CPP) using *therapy hours*, *number of caregiver visits*, and *duration of caregiver visits*. Latent variable modeling allows for the estimation of an unobserved, underlying construct (i.e., CPP) through observed variables (Bartholomew et al., 2011; Rabe-Hesketh et al., 2007). One loading on the latent variable was set to 1 to provide a metric for the latent variable.

The goodness-of-fit test for the MSEM was $X^{2}\left(42\right)=156.4$, significant with *p* < 0.001. This means that the hypothesized model does not fit the data perfectly. However, due to the test’s sensitivity to sample size and multivariate non-normality, we consulted alternative fit statistics. The RMSEA was 0.030, which indicates a close fit. The TLI (0.96) and CFI (0.98) were larger than 0.90, indicating good model fit. Lastly, the SRMR was 0.028, which also suggests that the model fit the data well. Overall, the model fits the data adequately. The estimates are interpreted below.

The final model predictors were self-reported gender, adoption status, caregiver effort, “*I belong here*”, FAD (family functioning), and caregiver program participation. For each categorical predictor, the researchers assigned a reference category against which the other categories were assessed. The reference categories were: “*male*” for self-reported gender, “*no*” for adoption, and “*exceptional*” for caregiver effort. As such, the interpretation of each categorical variable was a comparison to the reference category. For example, adoption results were reported as “*compared to those who are not adopted*, *adopted adolescents….*” For non-categorical variables, results should be read as for any regression.

## 5. Results

### 5.1. WT Outcomes

In order to answer the research question, “does caregiver engagement in adolescent WT foster improved outcomes”, researchers first conducted analyses to determine the mental health outcomes of adolescents in WT. Results indicate adolescents in WT reported improved mental health from pre- to post-data collection. All six YOQ-SR subscales (critical items, intrapersonal distress, interpersonal relationships, somatic, social problems, and behavioral dysfunction) showed statistically significant improvements (*p* < 0.001) from pre-treatment to post-treatment (see Table 2). Effect sizes ranged from medium to large (Cohen’s *d* between 0.41 and 0.73), which suggests substantial change in various aspects of adolescent mental health.

### 5.2. Caregiver Engagement

Next, researchers examined whether adolescent mental health outcomes were predicted by caregiver engagement, given the influence of other factors known to impact adolescent WT outcomes. The other factors known to affect adolescent WT outcomes were gender, adoption status, treatment belongingness, and family functioning. These factors were included in the structural model.

Number of caregiver visits, family therapy hours, and duration of caregiver visits significantly predicted the caregiver program participation latent variable (see Table 3). The unstandardized loadings (λ) should be interpreted such that a one-unit increase in the caregiver program participation latent variable predicted an 18.2 difference in therapy hours and a 5.04 difference in duration of visits. This finding suggests that if the caregiver program participation latent variable score were higher, the number of family therapy hours and duration of caregiver visits would likewise increase.

The latent variable caregiver program participation significantly predicted the YOQ subscales of critical items, intrapersonal distress, interpersonal relationships, and social problems. For example, a one-unit increase in the caregiver program participation latent variable predicted a 0.23 Reliable Change Index improvement in the YOQ subscale critical items.

Caregiver effort, as rated by clinicians, showed a lack of significant difference across all levels of the predictor. This may be explained by the small group sizes in the categories *none* and *low*. There were, however, significant differences between *exceptional* and *moderate* caregiver effort: *moderate* caregiver effort predicted lower mean change than *exceptional* parent effort on all YOQ subscales except Somatic issues. *High* caregiver effort differed from *exceptional* caregiver effort only in the YOQ social problems subscale, with analyses showing that *high* caregiver effort predicted a lower mean change than *exceptional* caregiver effort on the YOQ social problems subscale. These findings indicate that when controlling for other factors known to influence client change, caregiver effort plays a substantial role in client improvement.

### 5.3. Factors Affecting Adolescent WT Outcomes

The factors known to influence WT adolescent outcomes and thus included in the model were gender, adoption status, treatment belongingness, and family functioning. In terms of gender, females had a higher mean change than males across all YOQ subscales (see Table 4). This finding indicates that females reported greater mental health symptom reduction compared to males by the end of their WT experience. The magnitude of change was greatest on the YOQ subscale of intrapersonal distress and lowest on the YOQ subscale of behavioral dysfunction.

Adopted adolescents, on average, had lower mean change than non-adopted adolescents on all YOQ subscales except social problems. Across the remaining outcomes, the magnitude of change was greatest on the intrapersonal distress. This finding suggests that non-adopted adolescents reported greater improvement in mental health from pre- to post-WT than adopted adolescents.

The item “*I belong here*” predicted positive health change on all YOQ subscales. For example, controlling for all other predictors and holding facility ID constant, adolescents who indicated a one-unit higher “I belong here” at admission predicted the improvement of the YOQ subscale critical items Reliable Change Index by 0.03 over the course of treatment.

Family functioning at the time of admission was a significant predictor of mental health outcomes of WT clients. Across all YOQ subscales, the higher the family functioning score at admission, the greater the expected adolescent mental health improvement. For example, the amount of improvement in the YOQ subscale critical items is expected to be greater by 0.24 Reliable Change Indices for each one-unit higher family functioning score at admission.

Age also predicted mental health improvement on all YOQ subscales. For example, 0.04 Reliable Change Index more change in the YOQ subscale critical items is predicted for every one year older at which adolescents were admitted to the program.

## 6. Discussion

Findings from the present study clearly answer the study question. The study found that the variable “caregiver program participation” significantly predicted adolescent mental health outcomes, suggesting that the more caregivers were involved in WT family interventions, the more likely their adolescent child was to improve in the WT program. The study also found that greater caregiver effort predicted greater mean change in adolescent mental health outcomes of WT.

These findings implicate the strong relationship between adolescent WT outcomes and familial engagement in treatment, a relationship that may be explained most clearly using family systems theory. Using this theoretical lens, adolescent symptomology emerges within the context of the family system and links to whole family functioning (Ferenchick & Rosenthal, 2019; Yahav & Sharlin, 2002). From a family systems perspective, adolescents’ symptoms, such as oppositionality, have meaning within the family context (for example, “pay attention to me!”). Thus, adolescent symptoms may be understood by examining and treating family dynamics. From this perspective, stronger caregiver engagement in treatment is critical to optimal adolescent outcomes in WT.

Analyses showed that the variable caregiver program participation significantly predicted adolescent mental health outcomes in terms of critical items (such as suicidal ideation, self-harm, hallucinations, etc.), intrapersonal distress (depression, anxiety, fearfulness, etc.), interpersonal relationships (difficulty with relationships), and social problems (behaviors such as aggression, defiance, and conflict). These four areas represent a broad spectrum of adolescent functioning and mental health, suggesting that caregiver engagement in adolescent WT correlates with adolescent improvement in WT across a wide range of outcomes. This finding aligns with family systems theory, which emphasizes the interconnectedness and interdependent nature of family members, explaining how caregiver engagement and involvement in treatment can influence adolescent outcomes. This finding is congruent with existing research suggesting linkages between adolescent treatment outcomes and family engagement in that treatment (Coll et al., 2018; Liermann & Norton, 2016).

Studies of outpatient adolescent treatment help explain the link between parent involvement and adolescent treatment (Israel et al., 2007; Ugarte & Hastings, 2023). Parental involvement in adolescent treatment correlates with improvements in adolescent anxiety, academic performance, aggressive social media use, and internalizing symptoms of ADHD, among other issues (Corcoran & Dattalo, 2006; Lebovitz et al., 2020; Martins et al., 2019; Tan et al., 2019). In one study of outpatient CBT for adolescents with anxiety, caregiver engagement linked to both decreased caregiver interference with treatment progress and increased caregiver satisfaction with the treatment their adolescent received (Cardy et al., 2020). These findings align with family systems theory, which emphasizes the influence of systemic dynamics on individual behavior and well-being. From this perspective, caregiver involvement not only supports adolescent improvement but also helps shift family patterns to help foster a most supportive environment for recovery. Even when controlling for socioeconomic status, stronger parental involvement is linked to improvements in adolescents’ academic behaviors (Tan et al., 2019).

The present study also found several other important results. Notably, the higher the family functioning score at admission, the more significant the adolescent mental health improvement. This finding suggests that when adolescents reported stronger family functioning at intake, they were more likely to improve in WT. Thus, interventions to enhance family functioning and communication at home, prior to adolescent WT, may be critical in order to enhance WT outcomes for adolescents.

Adolescents in the present study who reported one unit higher “*I belong here*” at admission predicted the improvement of the critical items Reliable Change Index by the end of WT. This finding suggests that adolescents who believe they belong in treatment are more likely to improve in WT, a finding that is congruent with existing research suggesting that adolescents’ readiness to change links to stronger adolescent WT outcomes (Bettmann et al., 2013). WT programs should consider assessing adolescents’ and family members’ readiness to change at the start of treatment and focusing therapeutic work on whole family motivation for treatment. From a family systems perspective, increasing family members’ readiness to change may affect adolescents’ treatment outcomes (Miller et al., 2016). In addition, some have explored the importance of increasing youth voice in the treatment process (Pringle et al., 2023). Working collaboratively with adolescents and families to support the whole family in the transition to wilderness treatment is a key area.

### 6.1. Implications for Wilderness Therapy Programs

Family systems theory offers a useful lens through which to consider the findings from the present study. Most family systems therapists reject the idea of an *identified patient* who is solely to blame for the issues in the family (Ferenchick & Rosenthal, 2019). Instead, the most symptomatic family member acts as an emissary between the family and the rest of the world—a cry for help for the larger family dynamic (Ferenchick & Rosenthal, 2019). In adolescent WT, temporarily removing the adolescent from the home and treating them in WT may seem to solve the family’s problems and that time away may be meaningful for youth (Tucker et al., 2023). However, WT programs that treat the entire family system as the client and more directly engage the caregivers in the treatment process increase the likelihood of positive treatment outcomes. WT should consider facilitating more frequent family therapy through videoconference or in-person family therapy sessions so that family communication, emotional patterns, and other systemic issues are addressed.

In order to engage caregivers in adolescent treatment, WT programs could consider utilizing a systems approach such as Emotion-Focused Family Therapy (Foroughe et al., 2019; Lafrance et al., 2020; Quinn et al., 2023). This family therapy approach uses attachment theory as its lens, understanding adolescents’ symptoms and emotional distress as adaptive responses to insecurity in parent-adolescent relationships (Stavrianopoulos et al., 2014).

Emotion-Focused Family Therapy focuses on engaging and retaining caregivers in the treatment process, with the underlying belief that caregivers are as important in the therapeutic process as the patient. Therefore, caregivers are taught to regulate their own emotions, encouraged to release any shame or blame they may feel over the patient’s issues, and encouraged to view incremental change as progress. Many interventions in Emotion-Focused Family Therapy aim to engage caregivers in the treatment process by providing them with skills to emotionally support their adolescent child (Lafrance et al., 2020). Emotion-Focused Family Therapy includes addressing “caregiver blocks” in the treatment process and focuses on emotional expression and connectedness; thus, it may be ideal for adolescents and caregivers who have high levels of alienation and conflict with each other.

### 6.2. Limitations of the Study

The present study examined mental health outcomes only from adolescent reports. Thus, findings represent adolescents’ perspectives on their own mental health symptoms—not their caregivers’ perspectives. Additionally, the present study only utilized a sample of 12 WT programs, so findings from the present study should not be generalized to all WT programs or all adolescent treatment.

Without a comparison group in the present study, it is unclear if the findings reported are due to the results of the treatment; findings could be due to adolescent maturation and/or the specific contributions of program staff or specific interventions. While research shows that adolescents in WT sustain change after leaving treatment (DeMille et al., 2018), the present study did not examine adolescent functioning post-treatment, limiting our understanding of the lasting nature of these changes.

## 7. Future Research

The finding that caregiver engagement is an important factor in the outcomes of adolescent WT is compelling and merits further exploration. Future research should explore which family therapy interventions have the greatest effect on outcomes. Such research could help to define best practices in caregiver engagement and family therapeutic work in adolescent out-of-home treatment. Future research also should explore the relationship between other family therapy interventions and adolescent WT outcomes in order to determine the contribution of specific family interventions to outcomes.

Future research should include caregivers’ perspectives. Adolescents’ reports of improvements in family functioning do not always match their caregivers’ reports; additionally, those reports show a perception divide between caregivers of different genders (Tucker et al., 2016a). These disparities are worth exploring to understand whether discrepancies between adolescent experience and caregiver reports are factors in adolescent outcomes.

Within WT research, researchers primarily examined gender as a binary, explicitly identifying it as either male or female. WT programs have varying efficacy when outcomes are stratified by gender identity, a finding supported in the present study. Data for the current study included too few clients in the gender-diverse category to stratify the variable further. But future research should explore WT outcomes identifying adolescents with a range of self-chosen gender identities (Tucker et al., 2020).

## 8. Conclusions

The present study showed the strength of caregiver involvement and engagement in adolescent WT, as well as the importance of considering family functioning in adolescent treatment. This study suggests the importance of enhancing familial interventions in adolescent WT in order to improve outcomes. Given findings from this study, WT programs should consider expanding the ways that they involve families in treatment in order to optimize adolescent outcomes.

## Figures and Tables

**Table 1:** Family interventions utilized by WT programs and frequencies reported by program therapists.

Family Therapy Interventions	*N* Utilized This Intervention	Percentage of the Sample Reporting Use
**Total Sample** = 3220		
**Virtual Therapy**		
Telephonic family therapy	2115	66%
Video family therapy	1264	39%
**Targeted Family Therapy**		
Therapeutic on-site visits (on-site family therapy)	1660	52%
Therapy including other important family members	786	24%
Multi-family therapy	729	23%
Parent coaching	1934	60%
Exploring the family story/experience (i.e., family sculpture, genograms, writing the family story, evaluating family roles)	1619	50%
Home structure/rules evaluation	1517	47%
**Time Together During Program**		
Recreational on-site visits (on-site family therapy)	430	13%
Family pass near campus with therapeutic goal	111	3%
Family pass without therapeutic goals	30	1%
Family home pass with supervision/professional support	14	0.4%
Family home pass without supervision/professional support	61	0.02%
**Skill Building**		
Psychoeducational trainings for caregivers	2071	64%
Communication training/skill building for caregivers	2444	76%
Family seminars/support groups	1218	38%
**Writing Letters**		
Impact/intervention letters between caregiver and adolescent child in WT	2569	80%
Regular letter writing between caregiver and adolescent	3076	96%
Writing a family story	616	19%

**Table 2:** Mean subscale YOQ-SR RCI units and standard deviations pre- and post-treatment.

YOQ Subscale	Treatment		*t*-Value	*p*-Value	Mean Difference [95% CI]	Cohen’s *d* [95% CI]
	Pre-Mean (SD)	Post-Mean (SD)				
Critical Items	0.57 (1.00)	0.12 (0.86)	20.01	<0.001	0.45 [0.41, 0.49]	0.48 [0.43, 0.53]
Intrapersonal Distress	1.16 (1.53)	0.13 (1.27)	30.34	<0.001	1.03 [0.97, 1.1]	0.73 [0.68, 0.78]
Interpersonal Relationships	0.43 (0.93)	−0.07 (0.90)	25.63	<0.001	0.50 [0.46, 0.55]	0.55 [0.50, 0.60]
Somatic	0.45 (0.94)	0.08 (0.83)	19.31	<0.001	0.37 [0.33, 0.40]	0.41 [0.37, 0.46]
Social Problems	0.82 (1.15)	0.16 (1.05)	28.20	<0.001	0.66 [0.61, 0.71]	0.60 [0.55, 0.65]
Behavioral Dysfunction	0.40 (0.62)	0.08 (0.62)	21.68	<0.001	0.33 [0.29, 0.35]	0.53 [0.48, 0.58]

**Table 3:** Factor loadings and standard errors.

*η*	Predictor	λ (SE)	$\lambda^{*}$ (SE)	*p*-Value
Caregiver Program Participation	Number of Visits (*How many times did the adolescent’s caregivers visit during treatment*)	1	0.42 (0.04)	
	Therapy Hours (*On average*, *how long did the adolescent’s caregivers stay during each visit (hours)?*)	18.2 (1.89)	0.33 (0.03)	<0.001
	Duration of Visit (*While visiting*, *on average how many hours did the caregivers participate in therapy with their child?*)	5.04 (0.83)	0.93 (0.07)	<0.001

Note: η = latent variable; λ = unstandardized.

**Table 4:** Regression of YOQ-SR Reliable Change Index unit subscales on predictors controlling for facility ID.

	Critical Items	Intra-Personal Distress	Intra-Personal Relations	Somatic	Social Problems	Behavioral Dysfunction
Fixed Effects	B (SE)	B (SE)	B (SE)	B (SE)	B (SE)	B (SE)
Mean intercept	−0.33 *** (0.09)	−0.25 (0.13)	−0.51 *** (0.09)	0.07 (0.08)	−0.45 *** (0.11)	−0.18 ** (0.06)
Gender. *Reference Category “Male” (n = 1996)*						
Female (*n* = 1099)	0.34 *** (0.04)	0.45 *** (0.06)	0.19 *** (0.04)	0.14 *** (0.04)	0.18 *** (0.05)	0.09 *** (0.03)
Gender diverse (*n* = 290)	0.50 *** (0.07)	0.70 *** (0.1)	0.26 *** (0.07)	0.21 ** (0.06)	0.02 (0.08)	0.15 ** (0.04)
Adoption. *Reference Category “Not Adopted”* (*n* = 2475)						
Yes (*n* = 610)	−0.16 ** (0.05)	−0.4 *** (0.07)	−0.13 ** (0.05)	−0.2 *** (0.05)	−0.09 (0.06)	−0.12 *** (0.03)
Caregiver Effort. *Reference Category “Exceptional Caregiver Effort”* (*n* = 370)						
None (*n* = 6)	0.31 (0.5)	−0.02 (0.69)	−0.36 (0.5)	−0.03 (0.4)	−0.1 (0.57)	−0.16 (0.3)
Low (*n* = 196)	−0.07 (0.09)	−0.17 (0.12)	−0.18 (0.09)	−0.09 (0.08)	−0.16 (0.1)	−0.04 (0.06)
Moderate (*n* = 987)	−0.12 * (0.05)	−0.17 * (0.07)	−0.2 *** (0.05)	−0.05 (0.05)	−0.17 ** (0.06)	−0.08 * (0.03)
High (*n* = 1626)	−0.09 (0.05)	−0.03 (0.07)	−0.08 (0.05)	0.02 (0.04)	−0.15 ** (0.06)	−0.05 (0.03)
“I belong here”	0.03 *** (0.01)	0.07 *** (0.01)	0.02 *** (0.01)	0.01 * (0.01)	0.05 *** (0.01)	0.03 *** (0.00)
FAD	0.24 *** (0.03)	0.36 *** (0.04)	0.39 *** (0.03)	0.09 *** (0.0 3)	0.40 *** (0.04)	0.16 *** (0.02)
Age ^a^	0.05 ** (0.01)	0.09 *** (0.02)	0.04 ** (0.01)	0.07 *** (0.01)	0.07 *** (0.02)	0.06 *** (0.01)
Caregiver Program Participation	0.23 *** (0.08)	0.25 * (0.12)	0.21 ** (0.08)	0.06 (0.07)	0.24 * (0.1)	0.03 (0.05)

Note: *** *p* < 0.001; ** *p* < 0.01; * *p* < 0.05; ^a^ Age was grand-mean centered.

## Data Availability

Restrictions apply to the availability of these data. Data were obtained from the Outdoor Behavioral Healthcare Center at the University of New Hampshire and were made available by Anita Tucker at the University of New Hampshire. Requests to access the dataset should be directed to anita.tucker@unh.edu.
